# The IL-6 signaling pathway contributes critically to the immunomodulatory mechanism of human decidua-derived mesenchymal stromal cells

**DOI:** 10.1016/j.isci.2024.109783

**Published:** 2024-04-18

**Authors:** Hyemin Na, Keon-Il Im, Nayoun Kim, Junseok Lee, Sojin Gil, Gi-June Min, Seok-Goo Cho

**Affiliations:** 1 Institute for Translational Research and Molecular Imaging, The Catholic University of Korea, Seoul, Republic of Korea; 2 Department of Biomedicine & Health Sciences, College of Medicine, The Catholic University of Korea, Seoul, Republic of Korea; 3 Department of Hematology, Seoul St. Mary’s Hematology Hospital, College of Medicine, The Catholic University of Korea, Seoul, Republic of Korea

**Keywords:** Health sciences, Immunology, Stem cells research

## Abstract

Human bone marrow-derived mesenchymal stromal cells (BM-MSCs) have been proposed as a treatment for graft-versus-host disease (GVHD), which is a major complication following allogeneic hematopoietic cell transplantation. However, clinical trials have not yielded good results, and human decidua-derived mesenchymal stromal cells (DSCs) have been proposed as an alternative. In addition, the mechanism by which DSCs exert their immunomodulatory effects is still unknown. We found that knockdown of IL-6 in DSCs reduced the expression of PD-L1 and PD-L2, which are known as classical immune checkpoint inhibitors. Expression of PD-L1 and PD-L2 was restored by adding recombinant IL-6 to the DSCs. When DSCs and IL-6-knockdown DSCs were administered as treatment in a murine GVHD model, the group receiving IL-6-knockdown DSCs had significantly higher mortality and clinical scores compared to the group receiving DSCs. Taken together, these data suggest that the IL-6 signaling pathway is a crucial contributor to the immunosuppressive capacity of DSCs.

## Highlights

• Human DSCs exhibit a higher expression of IL-6 than BM-MSCs
• Human DSCs exhibit contact-dependent immunomodulatory effects
• Human DSCs increase the expression of PD-L1 and PD-L2 via the IL-6 signaling pathway
• Reduction in IL-6 expression within DSCs diminishes immunomodulatory effects

## Graphical abstract

**Figure:**
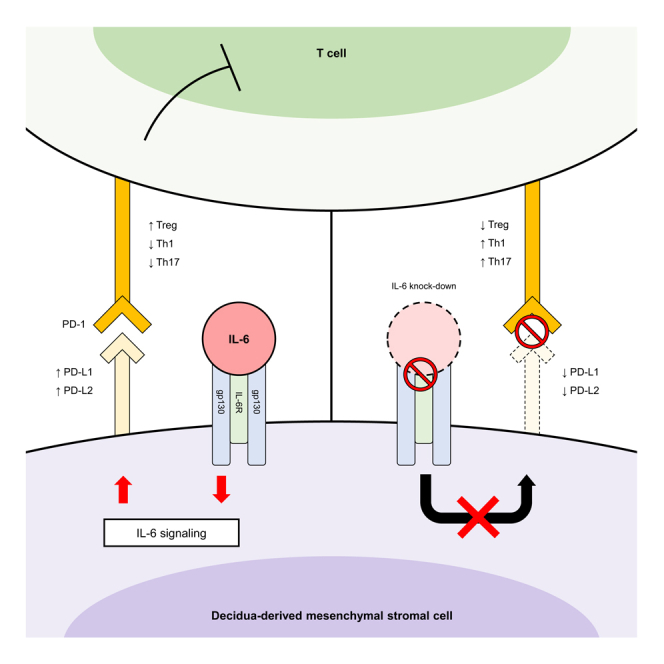

## Introduction

Allogeneic hematopoietic cell transplantation (allo-HCT) effectively treats both malignant and benign hematological diseases.^1^ However, side effects include graft-versus-host disease (GVHD) in 40%–60% of patients, which is associated with serious morbidity and mortality leading to poor clinical outcomes.^2^ The mortality rate can reach 15% caused by infections and persistent GVHD-related cytopenia with multi-organ disorders.^3^^,^^4^ In GVHD patients, alloreactive donor T cells view the histocompatibility antigens of host cells as foreign and produce inflammatory cytokines including tumor necrosis factor (TNF)-α, interleukin (IL)-6, and interferon (IFN)-γ.^5^ Although tacrolimus, cyclosporine, and steroids are clinically beneficial, the outcomes of acute GVHD patients who cannot tolerate steroids are poor and therefore require new treatments.^6^^,^^7^

Mesenchymal stromal cells (MSCs) have recently served as a cell-based therapeutic approach after transplantation.^8^ MSCs express low levels of the major histocompatibility complex II and suppress T cells despite the difference in major histocompatibility complex between the donors and recipient.^9^ The immunosuppressive effects are attributable principally to soluble factors including indamine 2,3-dioxygenase (IDO), transforming growth factor (TGF)-β, and prostaglandin E2 (PGE2).^10^^,^^11^^,^^12^ Through these effects, MSCs are capable of reducing allogenic T cell responses in GVHD patients.^13^ However, traditional bone marrow (BM)-derived mesenchymal stromal cells (BM-MSCs) require invasive harvesting; their differentiation and proliferation capacities decrease with age, and they are difficult to use in patients with genetic diseases.^14^^,^^15^ Thus, other adult and fetal tissues, including umbilical cord blood, amniotic fluid, and placental tissue, have been investigated as alternative stromal cell sources.^16^^,^^17^

Maternal and fetal immunity interact in the placenta and decidua during embryo implantation and pregnancy. In addition, previous studies have shown that DSCs exhibit higher xeno-immunosuppressive properties through PD-L1 and PD-L2 expression than BM-MSCs.^18^^,^^19^ Therefore, placental and decidual cells may exhibit intrinsic immunomodulatory properties and could also serve as a therapeutic approach for immune-mediated diseases. Decidua-derived mesenchymal stromal cells (DSCs) derived from fibroblast-like stromal cells that proliferate and differentiate are the major cells of human decidua and exhibit characteristics of stem cells.^20^ DSCs modulate both innate and adaptive immunity. DSCs express typical MSCs surface markers but do not produce the characteristic soluble factors of MSCs.^17^ In addition, previous studies have shown that DSCs exhibit higher xeno-immunosuppressive properties through PD-L1 and PD-L2 expression than BM-MSCs.^21^^,^^22^ Interactions between programmed death ligands and PD-1 in GVHD-affected tissues induce immune cell apoptosis and inhibition of T cell receptor (TCR)-mediated lymphocyte proliferation and pro-inflammatory cytokine secretion.^23^^,^^24^ Also, DSCs readily travel to target organs after intravenous injection because they are half the size of BM-MSCs.^25^^,^^26^ Together, the data suggest that DSCs might serve as effective immunosuppressors in GVHD patients. Several recent works have suggested that DSCs might usefully treat both acute and chronic GVHD.^27^^,^^28^ However, the mechanism by which DSCs induce immunosuppressive effects remains unknown.

We previously showed that umbilical cord MSCs that are similar to DSCs^29^ expressed higher levels of IL-6 than did BM-MSCs.^30^ IL-6 is a pro-inflammatory cytokine that induces endothelial permeability, cell recruitment, B cell maturation, and T cell survival.^31^^,^^32^^,^^33^ However, IL-6 also serves as an anti-inflammatory cytokine that exhibits immunosuppressive properties.^34^ In the latter context, IL-6 induces the production of IL-1 and TNF antagonists, promotes the secretion of IL-10 (a typical anti-inflammatory cytokine),^35^ and maintains the stability by catalyzing PD-L1 glycosylation in tumor microenvironments.^36^ In addition, IL-6 is thought to be important in terms of maintenance of stem cell stemness.^37^ We hypothesized that the PD-L1 and PD-L2 expression levels of DSCs were associated with IL-6 production, based on both the results of previous studies and the characteristic IL-6 actions.

In this study, we silenced IL-6 expression using a lentiviral-mediated short hairpin RNA (shRNA) to confirm the role played by IL-6 in DSC-mediated immunomodulation, and this silencing effect reduced the capacity of DSCs to inhibit T cell proliferation by decreasing PD-L1 and PD-L2 expression.

## Results

### Immunosuppressive effects of BM-MSCs and DSCs stimulated by IFN-γ or TNF-α

**Figure 1:**
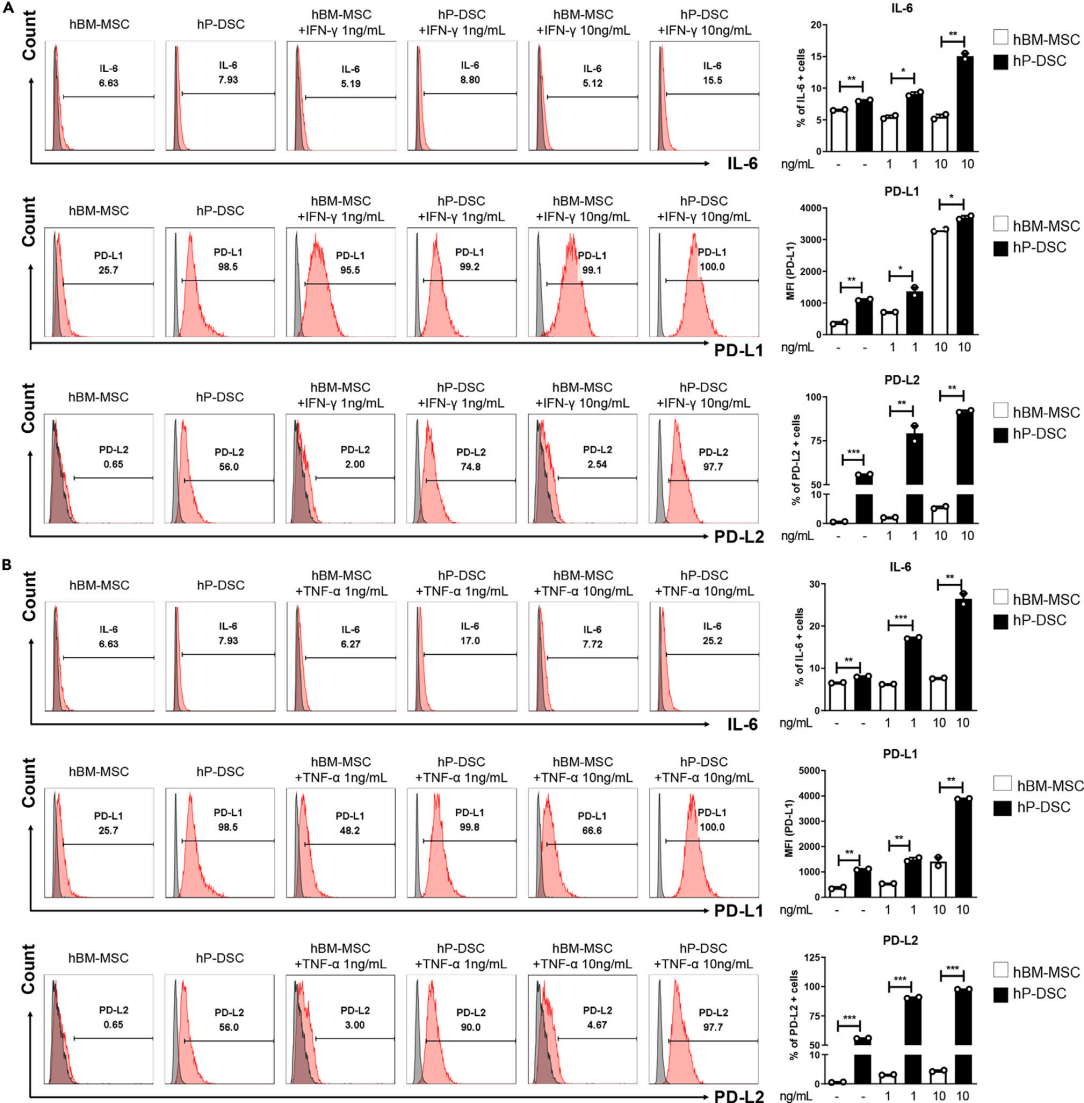
Immunosuppressive effects of BM-MSCs and DSCs stimulated with IFN-γ or TNF-α Expression levels of IL-6, programmed death-ligand (PD-L) 1, and PD-L2 revealed by flow cytometry of bone marrow-derived mesenchymal stromal cells (BM-MSCs) and decidua-derived mesenchymal stromal cells (DSCs) stimulated with 0, 1, or 10 ng/mL of (A) interferon (IFN)-γ or (B) tumor necrosis factor (TNF)-α for 48 h. (A) BM-MSCs exhibited increased PD-L1 expression only when treated with high concentrations of IFN-γ; IL-6 and PD-L2 had no effect. DSCs exhibited increased IL-6, PD-L1, and PD-L2 expression when treated with low or high concentrations of IFN-γ. (B) BM-MSCs were not very responsive to TNF-α, but DSCs exhibited increased levels of IL-6, PD-L1, and PD-L2. The columns represent the mean values of three independent experiments and the error bars represent standard deviations (SDs). ∗*p* < 0.05, ∗∗*p* < 0.01, ∗∗∗*p* < 0.001.

The immunosuppressive effects of MSCs are enhanced by the pro-inflammatory cytokine IFN-γ.^10^ Therefore, we created inflammatory conditions using different concentrations of IFN-γ and another pro-inflammatory cytokine, TNF-α, and measured IL-6, PD-L1, and PD-L2 expression levels in MSCs and DSCs. The stimulated MSCs and DSCs were analyzed by flow cytometry. PD-L1 and PD-L2 expression levels of both BM-MSCs and DSCs increased in a dose-dependent manner by addition of IFN-γ (Figure 1A) or TNF-α (Figure 1B). Notably, BM-MSCs reacted only to high concentrations of IFN-γ, whereas DSCs reacted to low levels of IFN-γ and TNF-α. However, BM-MSC IL-6 expression levels did not change with IFN-γ or TNF-α stimulation. Given that DSCs expressed significantly more IL-6 than did BM-MSCs under the same conditions, we considered that IL-6 might play a greater role in the immunosuppressive mechanism of DSCs than that of BM-MSCs.

### Lentivirus-mediated knockdown of IL-6 in DSCs

**Figure 2:**
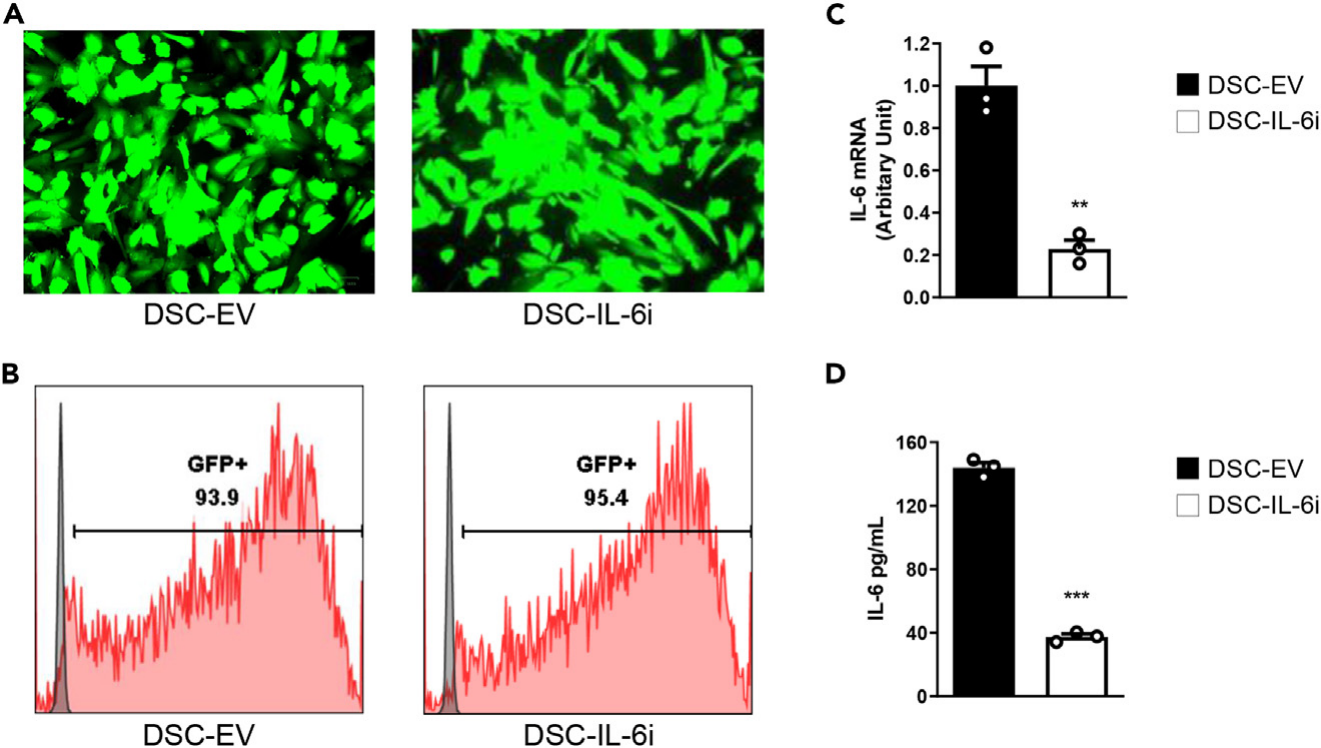
IL-6 shRNA transfection of DSCs (A) Cells were seeded into 12-well plates with Cellartis MSC Xeno-Free Culture Medium (Takara, Japan) and infected at a multiplicity of infection (moi) of 150/cell with GFP-expressing empty lentiviral vector (DSC-EV) or IL-6 short hairpin RNA (DSC-IL-6i). eGFP expression was evaluated 72 h later using a fluorescence microscope (scale bar: 400 μm). (B) eGFP expression was assayed via flow cytometry. The results of the three experiments were similar. (C) Quantification of IL-6-encoding mRNA via real-time qPCR. The expression levels were normalized to those of β-actin. (D) IL-6 secretion as determined by the human IL-6 Quantikine ELISA Kit. The DSC levels of IL-6 mRNA and the peptide decreased after shRNA transfection. The columns represent the mean values of three independent experiments and the error bars represent SDs. ∗*p* < 0.05, ∗∗*p* < 0.01, ∗∗∗*p* < 0.001.

We transduced to DSCs the GFP-expressing empty lentiviral vector (DSC-EV) or vectors with shRNAs targeting IL-6 (DSC-IL-6i). We employed three IL-6i vectors to avoid off-target effects. However, we show only one representative sequence. Fluorescence microscopy (Figure 2A) and flow cytometry (Figure 2B) showed that the transduction efficiencies exceeded 90%. IL-6i significantly reduced IL-6 mRNA production and secretion (Figures 2C and 2D).

### IL-6 knockdown reduced the capacity of DSCs to regulate T cell proliferation

**Figure 3:**
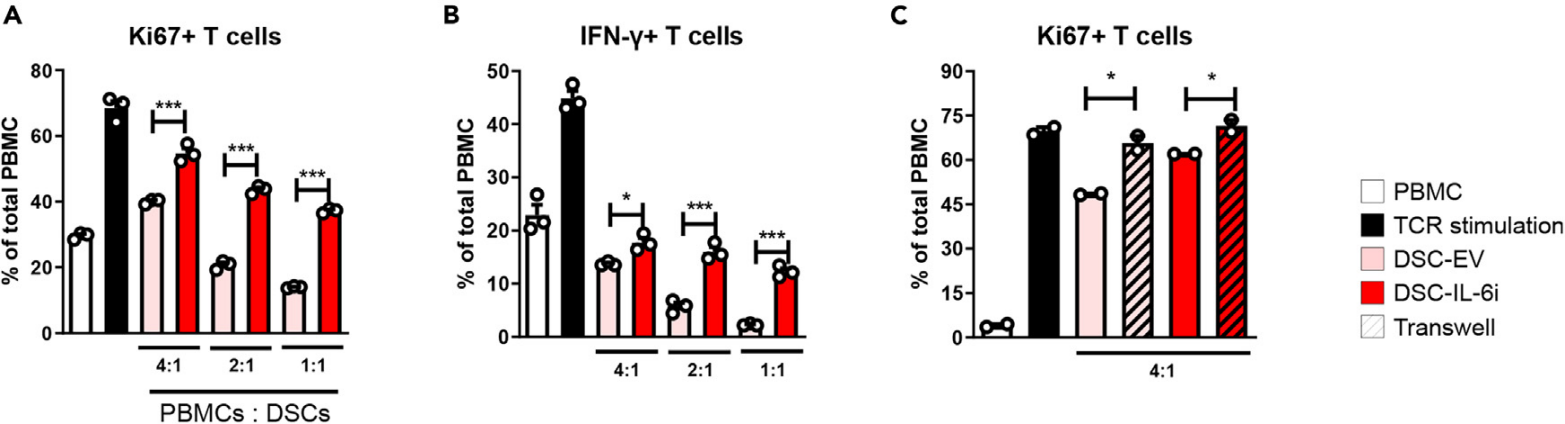
Co-culture of PBMCs with DSC-EV or DSCs-IL-6i cells at different PBMC/DSCs ratios (A and B) PBMCs were stimulated with 1 ng/mL of each of anti-CD3 and anti-CD28 and co-cultured with DSCs for 4 days. PBMCs were cultured in 96-well plates (105 cells/well); the ratios of PBMCs to DSCs were 4:1, 2:1, and 1:1. The percentages of (A) are proliferating T cells (Ki67+ cells among CD3^+^ cells) and (B) are activated T cells (IFN-γ+ cells among CD3^+^ cells) as revealed by flow cytometry. DSC-EV cells suppressed T cell proliferation in a dose-dependent manner, but DSC-IL-6i cells were less effective in this regard. (C) Transwell cell inserts were used to block cell-to-cell contact between DSCs and PBMCs; this abolished the immunosuppressive effect of DSCs. The columns represent the mean values of three independent experiments and the error bars represent SDs. ∗*p* < 0.05, ∗∗*p* < 0.01, ∗∗∗*p* < 0.001.

To explore whether T cells were suppressed by IL-6 secreted by DSCs, peripheral blood mononuclear cells (PBMCs) were isolated from healthy humans and a mixed lymphocyte reaction (MLR) was performed. PBMCs were stimulated with anti-CD3 and anti-CD28 antibodies and co-cultured with DSC-EV or DSC-IL-6i for 96 h, followed by flow cytometry analysis. The data indicate that DSC-EV inhibited T cell proliferation and reduced the number of activated T cells secreting IFN-γ. However, the regulation of T cell proliferation by DSC-IL-6i was significantly lower (Figures 3A and 3B). Next, we conducted a transwell experiment to determine whether cell-to-cell contact affected the capacity of DSCs to suppress T cell proliferation. Both DSC-EV and DSC-IL-6i exhibited reduced inhibitory capacities (Figure 3C), suggesting that the ability of DSCs to suppress T cell proliferation indeed required cell-to-cell contact.

### Levels of mRNA encoding PD-L1 and PD-L2 mRNA were decreased in DSC-IL-6i cells

**Figure 4:**
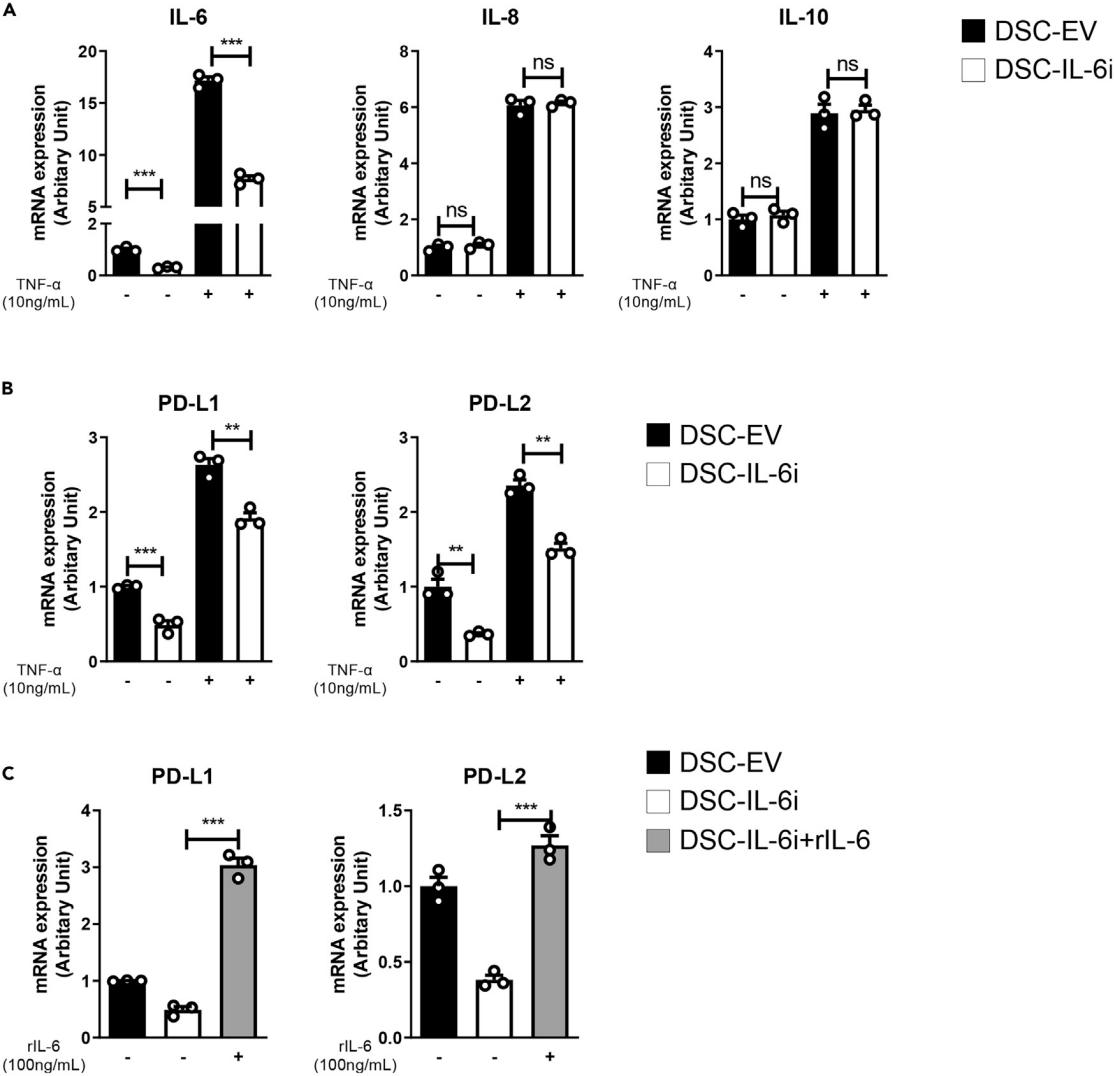
Gene expression by DSCs after IL-6 shRNA transduction (A) Quantitative PCR measuring IL-6, IL-8, and IL-10 levels. IL-6 shRNA transduction decreased the expression of only IL-6. DSC-EV and DSC-IL-6i cultures were treated with (B) TNF-α at 10 ng/mL, (C) recombinant human IL-6 (rIL-6) at 100 ng/mL, or untreated followed by culture for 48 h. DSC-IL-6i cells exhibited less PD-L1 and PD-L2 expression than controls, but TNF-α and rIL-6 rescued expression. The columns represent the mean values of three independent experiments and the error bars represent SDs. ∗*p* < 0.05, ∗∗*p* < 0.01, ∗∗∗*p* < 0.001.

We used quantitative reverse-transcription PCR (RT-qPCR) to measure the levels of mRNAs encoding IL-6, IL-8, and IL-10; the concentration of only mRNA encoding IL-6 was reduced in DSC-IL-6i cells (Figure 4A). We hypothesized that the contact-dependent immunomodulatory effects of DSCs were attributable to PD-L1 and PD-L2; both are well-known immune checkpoint molecules of the T cell-mediated immune system and promote immune tolerance by blocking the TCR-induced stop signal in a contact-dependent manner.^38^^,^^39^ Therefore, we explored if the levels of mRNAs encoding PD-L1 and PD-L2 varied by DSC IL-6 secretion status. Both mRNAs decreased following IL-6 knockdown, but addition of TNF-α or IL-6 to the culture medium rescued these reductions (Figures 4B and 4C). Thus, we observed that IL-6 could regulate PD-L1 and PD-L2 expression by DSCs.

### DSC IL-6 knockdown reduced GVHD treatment effectiveness

**Figure 5:**
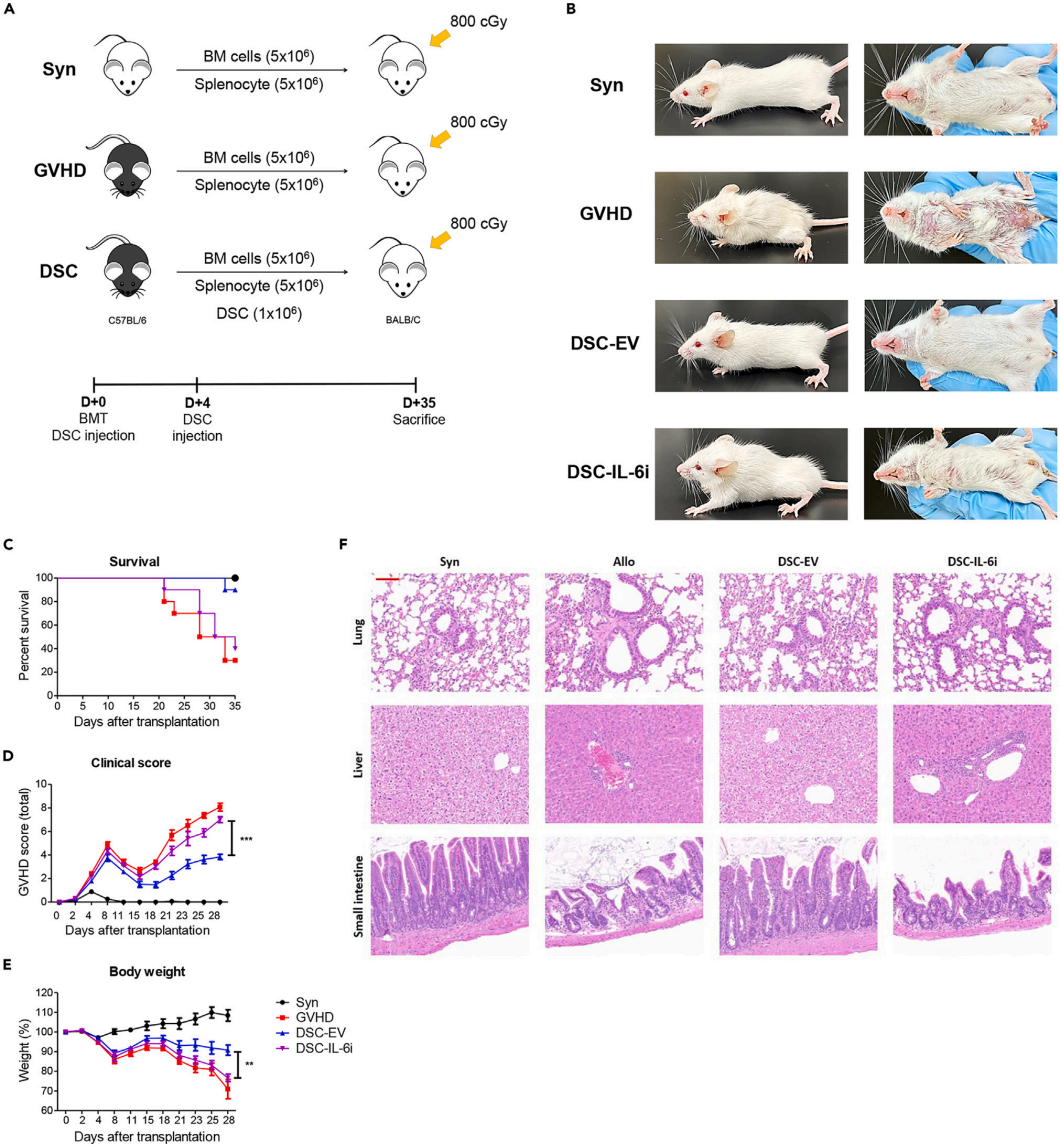
DSCs ameliorate GVHD severity (A) Recipient mice (BALB/c, H-2d) were irradiated with 800 cGy and intravenously injected with 5 × 10^6^ bone marrow cells and 5 × 10^6^ spleen cells from donor mice (C57BL/6, H-2b). DSC-EV (1 × 10^6^) and DSC-IL-6i (1 × 10^6^) mice were intravenously injected on days 0 and 4. (B) On day 25, after BMT, the DSC-EV-injected group was as healthy as the syngeneic transplantation (Syn) group. (C) Survival of GVHD mice after BMT. (D and E) Clinical GVHD scores were derived daily by assessing weight loss, posture, activity, fur texture, and skin integrity. The group injected with DSC-EVs significantly improved, but the group receiving DSC-IL-6i cells improved to a lesser extent. (F) Histological analyses of GVHD target organs, i.e., the lung (100×), liver (100×), and small intestine (100×).

Recipient mice (BALB/c, H-2d) were irradiated and received both BM and spleen cells from donor mice (C57BL/6, H-2d). At days 0 and 4, after BM transplantation (BMT), the mice were injected with DSC-EV or DSC-IL-6i (Figure 5A) and monitored daily thereafter in terms of survival, clinical GVHD scores, and body weight (Figures 5B–5E). The survival rates were 90% in the DSC-EV and 40% in the DSC-IL-6i groups (Figure 5C). The clinical scores and body weights of the two groups also differed significantly (Figures 5D and 5E). Histological analysis of GVHD target organs revealed the lack of any treatment effect in the DSC-IL-6i group. Compared to the DSC-EV group, the DSC-IL-6i group had immune cell infiltration in the lungs and liver, and the villi of the small intestine were shortened (Figure 5F). This suggests that IL-6 of DSC significantly affected the treatment of GVHD.

### DSC IL-6 knockdown reduced regulation of the T cell immune response in the GVHD mouse model

**Figure 6:**
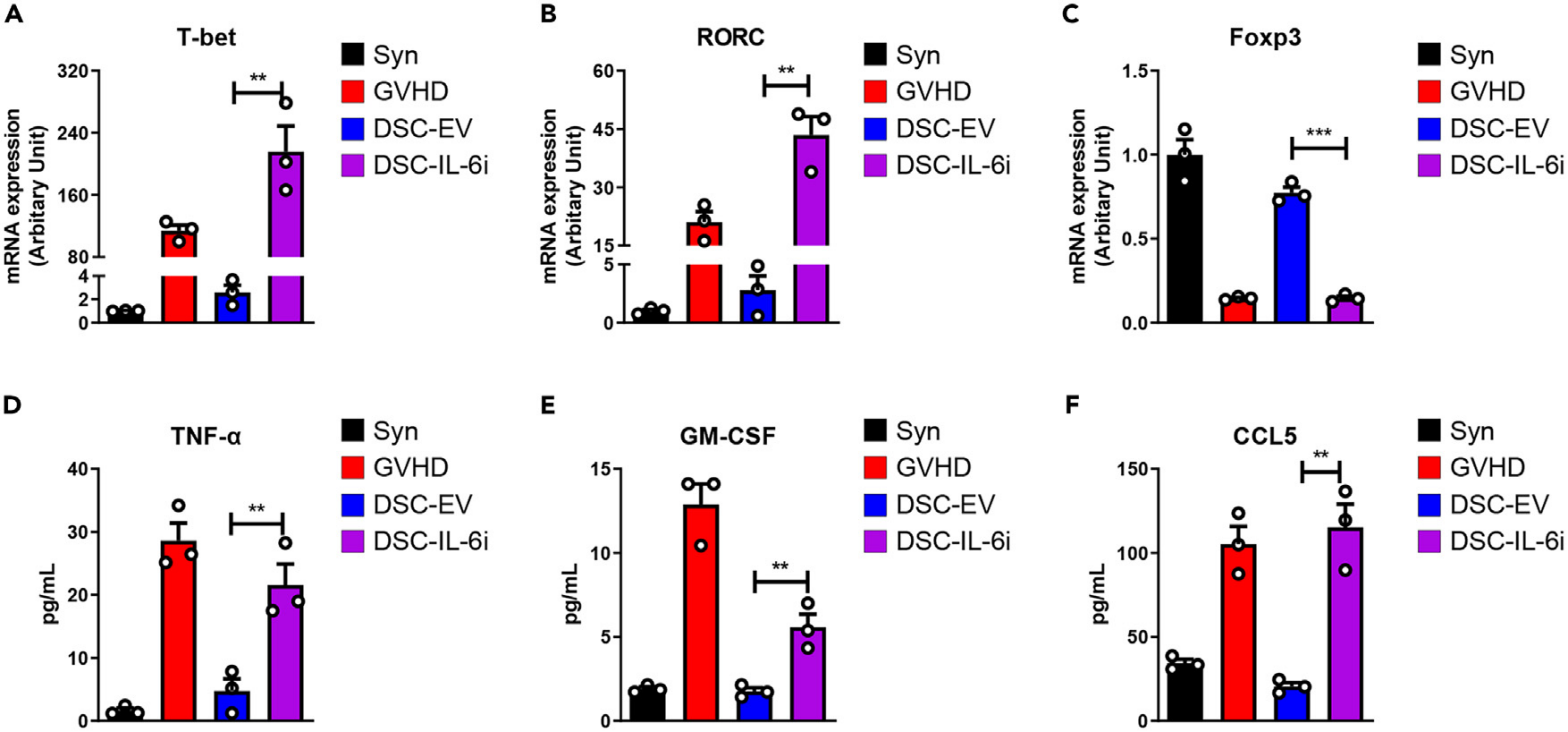
T cell-specific mRNA expression levels and cytokine secretion by mice with GVHD (A–C) Levels of mRNAs encoding (A) Th1, (B) Th17, and (C) the Treg cell transcription factor were determined via real-time PCR on day 35 after BMT. The control and DSC-EV-injected groups exhibited lower levels of mRNAs encoding T-bet and Rorc than did the other groups, but higher levels of mRNA encoding Foxp3. The GVHD group and the group injected with DSC-IL-6i exhibited the opposite behavior. (D–F) Serum levels of secreted inflammatory cytokines and chemokines measured via ELISA 35 days after BMT. The GVHD group and the DSC-IL-6i-injected group exhibited higher levels than did the other groups. The results are representative of those of two independent experiments. The columns represent the mean values of three independent experiments and the error bars represent SDs. ∗*p* < 0.05, ∗∗*p* < 0.01, ∗∗∗*p* < 0.001.

Th1 and Th17 cells play important roles in GVHD pathogenesis. A functional imbalance among Th1, Th17, and Treg numbers promotes GVHD progression.^40^^,^^41^^,^^42^^,^^43^ We performed *ex vivo* analysis of GVHD-induced mice. The levels of mRNAs encoding the T-box transcription factor TBX21 (T-bet), the retinoic acid receptor-related orphan receptor C (RORC), and the Forkhead box P3 (Foxp3) transcription factor of Th1, Th17, and Treg cells were measured in recipient spleens via real-time PCR. Compared to the GVHD group, the DSC-EV group exhibited reduced levels of mRNAs encoding T-bet (Figure 6A) and RORC (Figure 6B) but a higher level of Foxp3-encoding mRNA (Figure 6C). In contrast, no changes were apparent in the DSC-IL-6i group. We next determined the serum levels of cytokines and chemokines secreted by T cells. Compared to the DSC-EV group, the DSC-IL-6i group exhibited higher levels of TNF-α (Figure 6D), a representative inflammatory cytokine secreted by Th1 cells, and Granulocyte-macrophage colony-stimulating factor (GM-CSF) (Figure 6E), which contributes to the pathogenicity of Th17-mediated autoimmune diseases. Also, the level of C-chemokine ligand 5 (CCL5) that affects T cell homing was not reduced in the DSC-IL-6i group (Figure 6F), suggesting that IL-6 significantly influences the ability of DSCs to regulate the T cell immune response.

## Discussion

The use of MSCs to treat various immune-related diseases has been of interest by clinicians and researchers, and MSCs have been employed for many years to treat GVHD, which may be fatal following allo-HCT or organ transplantation. Initially, BM-MSCs, have been used, but the invasive nature of the procedure used to obtain BM-MSCs, and the fact that their immunosuppressive effects are activated by only through high concentrations of IFN-γ, indicate that new therapeutic approaches are required. In contrast, DSCs can be non-invasively obtained from placental tissue, proliferate rapidly, and are smaller in size compared to BM-MSCs.

Although DSCs share many characteristics with BM-MSCs, the mechanism by which they exert immunosuppressive functions is unique. Our results show that DSCs can be activated even by a low concentration of IFN-γ or other inflammatory cytokines, such as TNF-α, in contrast to BM-MSCs, which require priming by IFN-γ. These results suggest that DSCs may represent a cell therapy applicable to clinical GVHD patients who do not have high IFN-γ serum levels due to prolonged use of immunosuppressants.^44^^,^^45^^,^^46^^,^^47^ In addition, BM-MSCs mainly exhibit secretome-dependent immunomodulatory effects, whereas DSCs exhibit both cell-to-cell contact-dependent immunomodulatory effects. The cell-to-cell contact-dependent immunomodulatory effects of DSCs are consistent with the results of transwell experiments coculturing PBMCs with DSCs.

Previous studies reported higher PD-L1 and PD-L2 expression by DSCs than BM-MSCs, consistent with our results. In addition, we found that IL-6 secretion was significantly higher in DSCs than BM-MSCs. IL-6 is a classic inflammatory cytokine secreted by T cells and macrophages but also exhibits anti-inflammatory actions, potentially suppressing immunity via several mechanisms, including reductions in Th1 responses^48^^,^^49^^,^^50^ and promotion of immunosuppressive myeloid cell proliferation.^51^^,^^52^ In addition, IL-6 increases PD-L1 and PD-L2 expression by cancer cells in various tumor microenvironments.^36^^,^^53^^,^^54^ Therefore, we considered that the high PD-L1 and PD-L2 expression levels in DSCs might be related to IL-6.

We found that shRNA transfection of DSCs reduced IL-6 expression and significantly inhibited the T cell-suppressive capacity of such cells. The levels of mRNAs encoding PD-L1 and PD-L2 were reduced in IL-6-knockdown DSCs, but these decreases were rescued by TNF-α (which increases IL-6 signaling) or recombinant human IL-6 (rIL-6), supporting the idea that DSC PD-L1 and PD-L2 expression is related to IL-6 signaling. However, the underlying mechanism remains unclear and further study may be needed.

GVHD is a major complication of allo-HCT in which the intestines, liver, and lungs are damaged.^55^ We showed that DSC treatment of mice with GVHD significantly improved survival, clinical scores, and body weight and that downregulation of IL-6 expression by DSCs impaired these outcomes. Our results were supported by histological analyses of GVHD target organs. Thus, the potent immunomodulatory effects of DSCs are related to IL-6 signaling. Although high concentrations of IL-6 in GVHD patients indicate poor prognoses, the immunomodulatory effects of DSCs can be activated by IL-6 signaling, suggesting that DSCs may serve as a promising cell therapy for GVHD.

Th1 and Th17 cell-mediated responses can trigger fatal GVHD responses.^56^^,^^57^ In addition, donor T cell homing and migration contribute to GVHD development. Such homing/migration is regulated by the chemokine axis that includes CCL5/C-C chemokine receptor 5 (CCR5).^58^ Of the various cytokines secreted by Th17 cells, GM-CSF plays a particularly important role in terms of inducing inflammation and thus contributes to the pathogenicity of Th17-mediated autoimmune diseases.^59^^,^^60^ Induction of the Th1 and Th17 pathways suppresses Th2 and Treg cell actions and thus increases the severity of acute GVHD after allo-BMT. We found that the serum levels of CCL5, TNF-α, and G-CSF were higher in the group treated with IL-6-knockdown DSCs than in the group that received wild-type DSCs. The levels of mRNAs encoding T-bet and RORC were higher in the group treated with IL-6-knockdown DSCs, and the level of mRNA encoding Foxp3 was lower. These results suggest that IL-6 of DSCs is associated with downregulation of the Th1 and Th17 responses and that other (as yet unknown) mechanisms are involved in expansion of Treg cell numbers by IL-6. Together, the results suggest that the DSC T cell immune responses are regulated by IL-6 signaling.

In conclusion, DSCs exhibited a cell-to-cell contact-dependent immunomodulatory effect and expressed higher PD-L1 and PD-L2 levels than did BM-MSCs. Blocking of the DSC IL-6 signaling pathway abolished the immunomodulatory effects and PD-L1 and PD-L2 expression, suggesting that the immunomodulatory effects of DSCs are critically dependent on IL-6 signaling. In a murine GVHD model, DSCs improved survival, clinical scores, and weight loss, but IL-6-knockdown DSCs did not. Together, our findings suggest that the potent immunomodulatory effects of DSCs can be attributed to IL-6 signaling.

### Limitations of the study

We found that IL-6 in DSCs contributes critically to the immunomodulatory mechanism. However, we have not clearly determined through which signaling pathway IL-6 enhances PD-L1 and PD-L2 expression and enhances immunomodulatory ability, which is a drawback of our study. We will further investigate through which signaling pathways IL-6 enhances immunosuppressive capacity in DSCs in follow-up studies.

## STAR★Methods

### Key resources table

**Table:** 

REAGENT or RESOURCE	SOURCE	IDENTIFIER
**Antibodies**		

Human IL-6	eBioscience™	Cat#12-7069-81; RRID:AB_466167
Human CD273 (B7-DC, PD-L2)	BioLegend	Cat#345506; RRID:AB_2161994
Huamn CD274 (PD-L1)	BDBiosciences	Cat#558017; RRID:AB_396986

**Bacterial and virus strains**		

HIT Competent Cells-DH5alpha Value 108	RBC	Cat#RH617
psPAX2	addgene	Cat#12260
pMD2.G	addgene	Cat#12259
IL6 Human shRNA Plasmid Kit	Origene	Cat#TR312162

**Chemicals, peptides, and recombinant proteins**		

Recombinant human IFN-γ	PeproTech	Cat#300-02
Recombinant human TNF- α	PeproTech	Cat#300-01A
Recombinant human IL-6	PeproTech	Cat#200-06
SYBR Green Supermix	BIO-RAD	Cat#1708882
TurboFectin 8.0	Origene	Cat#TF81001

**Critical commercial assays**		

NucleoBond Xtra Midi EF	Macherey-nagel	Cat#740420
RNeasy Mini Kit	Qiagen	Cat#74104
qPCR RT Master Mix with gDNA Remover	TOYOBO	Cat#FSQ-301
Human IL-6 Quantikine ELISA Kit	R&D system	Cat#D6050B

**Experimental models: Cell lines**		

DSCs	Human placenta	Not from commercial sources or biological repositories
PBMCs	Human peripheral blood	Not from commercial sources or biological repositories

**Oligonucleotides**		

hβ-actin F: AGCTACGAGCTGCCTGAC	Davies et al.^61^	https://pubmed.ncbi.nlm.nih.gov/27671847/
hβ-actin R: AAGGTAGTTTCGTGGATGC	Davies et al.^61^	https://pubmed.ncbi.nlm.nih.gov/27671847/
hIL-6 F: AGACAGCCACTCACCTCTTCAG	Origene	Cat#HP200567
hIL-6R: TTCTGCCAGTGCCTCTTTGCTG	Origene	Cat#HP200567
hPD-L1 F: GGCATCCAAGATACAAACTCAA	Davies et al.^61^	https://pubmed.ncbi.nlm.nih.gov/27671847/
hPD-L1 R: CAGAAGTTCCAATGCTGGATTA	Davies et al.^61^	https://pubmed.ncbi.nlm.nih.gov/27671847/
hPD-L2 F: GAGCTGTGGCAAGTCCTCAT	Davies et al.^61^	https://pubmed.ncbi.nlm.nih.gov/27671847/
hPD-L2 R: GCAATTCCAGGCTCAACATTA	Davies et al.^61^	https://pubmed.ncbi.nlm.nih.gov/27671847/
hIL-8 F: GAGAGTGATTGAGAGTGGACCAC	Origene	Cat#HP200551
hIL-8 R: CACAACCCTCTGCACCCAGTTT	Origene	Cat#HP200551
hIL-10 F: TCTCCGAGATGCCTTCAGCAGA	Origene	Cat#HP200540
hIL-10 R: TCAGACAAGGCTTGGCAACCCA	Origene	Cat#HP200540
mT-bet F: CCACCTGTTGTGGTCCAAGTTC	Origene	Cat#MP216689
mT-bet R: CCACAAACATCCTGTAATGGCTTG	Origene	Cat#MP216689
mRorc F: GTGGAGTTTGCCAAGCGGCTTT	Origene	Cat#MP212434
mRorc R: CCTGCACATTCTGACTAGGACG	Origene	Cat#MP212434
mFoxp3 F: CCTGGTTGTGAGAAGGTCTTCG	Origene	Cat#MP204954
mFoxp3 R: TGCTCCAGAGACTGCACCACTT	Origene	Cat#MP204954

### Resource availability

#### Lead contact

Further information and requests for resources and reagents should be directed to and will be fulfilled by the lead contact, Dr. Seok-Goo Cho (chosg@catholic.ac.kr).

#### Materials availability

This study did not generate new materials.

#### Data and code availability

• All data can be obtained from the lead contact, provided the request is reasonable.
• This paper does not report original code.
• Any additional information required to reanalyze the data reported in this paper is available from the lead contact upon request.

### Experimental model and study participant details

#### Mice

C57BL/6 (H-2b) and BALB/c (H-2d) mice 8–10 weeks of age were purchased from Orient Bio (Seongnam, Korea) and kept under specific pathogen-free conditions in a facility with a humidity of 55 ± 5%, a 12-/12-h light/dark cycle, and a temperature of 22 ± 1°C. The air was HEPA-filtered to exclude bacteria and viruses. The animals had free access to mouse chow and tap water. All recipient BALB/c H-2d mice were exposed to 800 cGy of radiation delivered by a Mevatron MXE-2 instrument (Siemens, New York, USA) at 70 cGy/min. In the GVHD group (n = 13), mice were injected intravenously with 5 × 106 BM cells and 5 × 106 spleen cells from donor mice (C57BL H-2b). Mice in the control group (Syn group, n = 10) were similarly irradiated, but the donor BM and spleen cells were obtained from BALB/c H-2d mice; GVHD was not induced. Survival was monitored daily after BM transplantation (BMT), and the extent of clinical GVHD assessed weekly by scoring changes in weight, posture, activity, fur texture, and skin integrity. The animal experiments were approved by the Institutional Animal Care and Use Committee of the School of Medicine, Catholic University of Korea, in line with the Laboratory Animals Welfare Act.

#### Primary cell cultures

The study has been approved by the School of Medicine, Catholic University of Korea Institutional Review Board. Human placentas were obtained from healthy mothers who were seronegative for HIV; hepatitis A, B, and C; syphilis; HTLV 1-/2; and CMV after obtaining informed consent. The fetal membrane was carefully separated from the placenta and washed in phosphate-buffered saline (PBS). An equal volume of trypsin/EDTA was added to the chopped tissue for 10 min at 37°C and then discarded. The tissues were then incubated twice in trypsin/EDTA for 40 min at 37°C, and the trypsin digest was collected and washed in medium. The digested cells were seeded, and when cells in the trypsin-digested suspension had grown to 90% confluence, they were harvested and inoculated into fresh flasks containing medium. DSCs expressed CD166, CD105, CD73, CD44, and CD29 but not the hematopoietic markers CD34, CD14, and CD45. All DSCs were negative for bacteria, mycoplasma, and fungi prior to infusion. All DSCs were cultured and expanded in a laboratory that followed good manufacturing practice.

Peripheral blood was obtained from healty donor. Peripheral blood mononuclear cells (PBMCs) were isolated by Ficoll density gradient centrifugation. Cells were cultured and activated in the presence of anti-CD3/CD28 in Roswell Park Memorial Institute (RPMI) 1640 medium (Gibco, New York, USA) with 20 mM 2-(4-(2-hydroxyethyl)piperazin-1-yl)ethanesulfonic acid (HEPES; Gibco), 2 mM L-glutamine, 5% (v/v) heat-inactivated fetal bovine serum, 100 mM sodium pyruvate, and 1% (w/v) antibiotics (penicillin (10 U/mL)–streptomycin (10 μg/mL)).

### Method details

#### Transduction of shRNAs

Human IL-6 shRNA was purchased from Origin (Maryland, USA). We used two different target sequences to avoid off-target effects (IL-6A: CCAACCACAAATGCCAGCCTGCTGACGAA, IL-6B: CCAGGAAGATCCAAAGATGTAGCC, IL-6C: GAGTAGTGAGGAACAAGCCAGAGCTGTGC; both 5ʹ to 3ʹ). The shRNA plasmid was transduced into 293T cells with an envelope plasmid (pMD.2G) and a packaging plasmid (psPAX2); after 72 h of culture, the supernatant was concentrated using a 5xLenti Concentrator. DSCs were infected with the concentrated culture supernatant at a multiplicity of infection of 100, and polybrene was added to 4 μg/mL with or without human recombinant TNF-α (PeproTech, New Jersey, USA).

#### Mixed lymphocyte reaction (MLR)

The mixed lymphocyte reaction (MLR) proceeded in Roswell Park Memorial Institute (RPMI) 1640 medium (Gibco, New York, USA) with 20 mM 2-(4-(2-hydroxyethyl)piperazin-1-yl)ethanesulfonic acid (HEPES; Gibco), 2 mM L-glutamine, 5% (v/v) heat-inactivated fetal bovine serum, 100 mM sodium pyruvate, and 1% (w/v) antibiotics (penicillin (10 U/mL)–streptomycin (10 μg/mL)). Peripheral blood mononuclear cells (PBMCs) (1 × 10^5^/well) from a healthy donor were stimulated with anti-CD3 and anti-CD28 antibodies and irradiated (2,000 rad); DSCs were then added. Cultures were maintained at 37°C under 5% (v/v) CO_2_ and subjected to flow cytometry after 5 days.

#### Enzyme-linked immunosorbent assay (ELISA)

IL-6 levels were measured using a sandwich ELISA. Anti-human IL-6 (R&D Systems, Minneapolis, USA) was added to a 96-well plate (Nunc, Roskilde, Denmark) and incubated overnight at 4°C. The wells were blocked with blocking solution (PBS with 1% (w/v) bovine serum albumin (BSA; Gibco) and 0.05% (v/v) Tween 20 (Bio-Rad, California, USA)) for 2 h at room temperature. The test samples and standard recombinant IL-6 (R&D Systems, Minnesota, USA) were added to separate wells of the 96-well plate, followed by incubation at room temperature for 2 h. The plate was washed, biotinylated IL-6 polyclonal antibody (R&D Systems) added, and the reaction proceeded for 2 h at room temperature. The plate was washed, 2,000-fold diluted ExtrAvidin-alkaline phosphatase (Sigma-Aldrich, Missouri, USA) added, and the reaction proceeded for a further 2 h. The plate was washed and 50 μL of p-nitrophenyl phosphate disodium salt (Pierce Chemical Company, Illinois, USA) diluted to 1 mg/mL in diethanolamine buffer (Sigma-Aldrich) was added to each well. Absorbance was measured at 405 nm using an ELISA microplate reader (Molecular Devices, California, USA).

#### Real-time quantitative PCR

Total RNA was extracted using an RNeasy Micro Kit (Qiagen, Maryland, USA) and 2-μg amounts reverse-transcribed at 50°C for 2 min followed by 60°C for 30 min. Quantitative PCR was performed using iQ SYBR Green Supermix and a Real Time PCR CFX96 Touch platform (Biorad, California, USA) as instructed by the manufacturer. The crossing point (Cp) was the maximum second derivative of the fluorescence curve. Negative controls lacked template DNA. The relative mRNA expression levels were obtained by the ΔΔCt method; β-actin served as the internal control.

### Quantification and statistical analysis

Statistical analysis and graphing were performed using Prism 9.0. Statistical tests used for the specific analyses and the number of independent experiments are indicated in each figure legend. The columns represent the mean values of experiments and the error bars represent SDs. When data were analyzed by T-Test, p<0.05 was considered significant.
